# Nanocomplexes loaded with miR-128-3p for enhancing chemotherapy effect of colorectal cancer through dual-targeting silence the activity of PI3K/AKT and MEK/ERK pathway

**DOI:** 10.1080/10717544.2020.1716882

**Published:** 2020-02-24

**Authors:** Xin Liu, Chao Dong, Siping Ma, Yongpeng Wang, Tao Lin, Yanxi Li, Shihua Yang, Wanchuan Zhang, Rui Zhang, Guohua Zhao

**Affiliations:** a Department of Colorectal Surgery, Cancer Hospital of China Medical University, Liaoning Cancer Hospital & Institute, Shenyang, China;; b Department of the Second Medical Oncology, The 3rd Affiliated Hospital of Kunming Medical University, Yunnan Tumor Hospital, Kunming, China;; c Department of General Surgery, Cancer Hospital of China Medical University, Liaoning Cancer Hospital and Institute, Shenyang, China

**Keywords:** MicroRNAs, nanocomplexes, tumor-targeting, signaling pathways, 5-Fluorouracil

## Abstract

Although microRNAs (miRNAs)-based cancer therapy strategies have been proved to be efficient and superior to chemotherapeutic agents in certain extent, the unstable properties of miRNAs significantly impaired the wide application. Therefore, how to safely deliver the miRNAs to the targeted site of action is the most pivotal step to achieve the ideal treatment effect. In the present work, the miR-128-3p, which is able of inducing chromosomal instability, was loaded into the nanocomplexes developed by the PEG-PDMAEMA (PDMAEMA-NP). By this way, the miR-128-3p was shielded from exposure to various degrading enzymes in bloodstream. Additionally, the PEGylation endowed the PDMAEMA-NP with long time of circulation as demonstrated *in vivo* by pharmacokinetics investigation. To target and deliver the miR-128-3p to the site of action, a tumor-homing peptide CPKSNNGVC, which specifically targets the monocarboxylate transporter 1 (MCT1), was decorated on the surface of PDMAEMA-NP. Both *in vitro* and *in vivo* experiments demonstrated that more efficient delivery of miR-128-3p to cells or tumor tissues was obtained by the PDMAEMA-NP than plasmid. Additionally, modification of C peptides further enhanced the tumor accumulation of miR-128-3p, and in turn contributed to the stronger tumor growth inhibition effect. Underlying mechanisms study revealed that the miR-128-3p inhibited the growth, migration, and invasion of colorectal cancer (CRC) cells and progress of CRC tissues through silence of the activity of PI3K/AKT and MEK/ERK pathway. By this way, the chemotherapy effect of 5-Fluorouracil (5-Fu) was dramatically improved after co-treating the cells with miR-128-3p formulations.

## Introduction

As one of the prevalent cancer types, the colorectal cancer (CRC) has been the fourth leading cause of cancer-related mortality (Siegel et al., 2014). As previous statistical data revealed that nearly 1.4 million new patients were detected with CRC in a year and about 0.7 million death cases were reported due to the progress or relapse of CRC (Arnold et al., 2017; Araghi et al., 2019). Unfortunately, the concealed characteristics, late diagnosis, and rapid progression of CRC generally paved an obstacle in the achievement of satisfactory treatment effect (Qiu et al., 2016; Lee et al., 2017; Armelao et al., 2018). Additionally, the emergence of drug-resistance due to multiple mechanisms resulted in treatment failure in more than 90% patients (Rihawi et al., 2015).

As the most important small and non-coding regulatory molecules, microRNAs (miRNAs) are able of mediating a wide array of signaling pathways, which are responsible for a series of cellular process (Fabian & Sonenberg, 2012; Choi et al., 2013). However, the aberrant regulation of miRNA, mainly due to genetic or epigenetic mechanisms, was closely related to tumorigenesis, progression, invasion and metastasis of many cancer types (Popov et al., 2015; Petrovic et al., 2017). Furthermore, it was reported that counterbalancing the function of increased or decreased miRNAs has shown excellent anticancer effect (Liu et al., 2017). Among a series of miRNAs, miR-128-3p has been identified to speed up cell cycle arrest and chromosomal instability by suppressing the spectrin alpha and non-erythrocytic 1 (Zhang et al., 2016). It was also indicated that miR-128-3p was intimately associated with hepatocellular carcinoma (Guo et al., 2018) and acute lymphoblastic leukemia (Rzepiel et al., 2019).

However, the major challenge of using miRNA for cancer treatment is the low circulation half-life of naked synthetic small RNAs after they are injected into the bloodstream mainly due to the abundant nucleases (Fernandez-Piñeiro et al., 2017). A viable strategy to circumvent the rapid intravascular degradation is to shield the miRNAs by enclosing them into nanocarriers (Fernandez-Piñeiro et al., 2017). Additionally, nanocarriers can be designed to enhance the drug biodistribution, accumulation kinetics, and sustained release profile by decoration with tumor-homing peptides or aptamers (He et al., 2018). Such targeted delivery strategy was supposed to be able of offering the possibility to increase the therapeutic efficacy of intravenously administered anticancer formulations and minimizing adverse effects in healthy organs simultaneously (Xiao et al., 2018).

In the present study, a PDMAEMA nanocomplex loaded with miR-128-3p (PDMAEMA-NP) was designed for combating the progress of CRC. To achieve the goal of targeting delivery of miRNAs to tumor sites, a recently developed tumor homing-peptide CPKSNNGVC (C peptide for short) was further decorated on the surface of PDMAEMA-NP (CPDMAEMA-NP). The CPKSNNGVC was designed to specifically target the monocarboxylate transporter 1 (MCT1) which was overexpressed in series of CRC cells, such as the Caco-2, HCT 116, and HCT-15, while not the normal non-cancer cells (Ferreira et al., 2019). In this case, the developed CPDMAEMA-NP was supposed to be CRC-recognizable and stable enough to deliver the miR-128-3p to the sites of action.

## Materials and methods

### Materials

The carboxyl-poly(ethylene glycol)-poly (2-(N,N′-dimethylamino)ethylmethacrylate) (HOOC-PEG-PDMAEMA) and methoxy poly(ethylene glycol)-poly(2-(N,N′-dimethylamino)ethylmethacrylate) (MPEG-PDMAEMA) were purchased from Seebio Co., Ltd. (Shanghai, China). CPKSNNGVC-NH_2_ peptide was synthesized by Chinese Peptide Company (Hangzhou, China). Endocytosis inhibitors including chlorpromazine, Brefeldin A, NaN_3_, cytochalasin B, filipin, and monensin were purchased from Sigma (St. Louis, MO). 5-Fluorouracil (5-Fu) was purchased from Xi’an Sanjiang Biological Engineering Co. Ltd. (Xi’an, China). The miR-128-3p and corresponding negative control (NC) were obtained from Gene Pharma (Shanghai, China). The BCA^TM^ Protein Assay Kit was purchased from Pierce (Appleton, WI) while the polyvinylidene difluoride (PVDF) membrane was obtained from Millipore (Billerica, MA). The complete RPMI 1640 cell culture media supplemented with 10% fetal bovine serum (FBS) and 1% penicillin/streptomycin was purchased from Life Technologies (Carlsbad, CA).

### Cell culture and animal models

The normal colorectal epithelial cells HCoEpiC, and the CRC cell lines HT29, HCT 116, Caco-2, SW480 cells, and the drug-resistant HCT-15 were all achieved from the American Type Culture Collection (Manassas, VA). The cells were cultured in the complete RPMI 1640 media as mentioned above.

Male Sprague Dawley (SD) rats (200 ± 20 g) and the specific pathogen-free male BALB/c nude mice, age of 4–5 weeks and weight of 20 ± 2 g, were obtained from the BK Lab Animal Ltd. (Shanghai, China). For establishment of HCT-15 tumor-bearing mice model, 2 × 10^6^ cells were subcutaneously injected into the right flanks of mice. Then, the mice were raised under the standard conditions for further experiments. Of great importance, all animal experiments were performed in accordance with guidelines evaluated and approved by the ethics committee of Cancer Hospital of China Medical University.

### Preparation of nanocomplexes

The blend of MPEG-PDMAEMA and HOOC-PEG-PDMAEMA was dissolved by the diethyl pyrocarbonate-treated water at the ratio of 9:1. Then, the miRNA solution was added slowly followed by mixture for 30 s and incubation for 20 min at room temperature. Of great importance, the N/P ratio was set at 10:1. Subsequently, the formed nanocomplexes (PDMAEMA-NP) were obtained via centrifugation at 14,500 rpm for 1 h. For decoration with C peptides, the collected PDMAEMA-NP was resuspended by PBS and reacted with the C peptides at the molar ratio of 1:1.2 for 4 h at room temperature. Then, the peptides-functionalized nanocomplexes were collected through centrifugation (14,500 rpm for 45 min).

### Characterization of the developed nanocomplexes

The morphologies of the developed PDMAEMA-NP and CPDMAEMA-NP were respectively observed using the transmission electron microscope (TEM) (H-600, Hitachi, Tokyo, Japan). The particle size, zeta potential, and size distribution were determined using the dynamic light scattering detector (Zetasizer, Nano-ZS, Malvern, UK).

### Cell transfection

The miR-128-3p was amplified followed by colony into pcDNA3.1 Vector (GenePharma, Shanghai, China). Then, the Lipofectamine 2000 (Invitrogen, Carlsbad, CA) was used to realize the cell transfection in accordance with the guidelines. For cell transfection, 1 × 10^4^ cells were seeded in six-well plates and allowed to grow for 80% confluence. Then, the cells were respectively co-incubated with 10 μg of plasmid (pcDNA3.1 + NC or pcDNA3.1 + miR-128-3p) plus lipofectamine 2000, PDMAEMA-NP, and CPDMAEMA-NP for 6 h.

### Cellular uptake assay

After HCT 15 cells in the 96-well plates (5 × 10^3^ cells/per well) were grown to 80% confluence, different concentrations of FITC-labeled PDMAEMA-NP and CPDMAEMA-NP were respectively added in to each well. After co-incubation of cells with the NPs for 1 h, the old medium was removed and the cells were washed three times by PBS followed by fixation with 4% formaldehyde solution. Then, the fluorescent intensity was qualitatively determined under the fluorescent microscopy (Leica DMI4000 B, Wetzlar, Germany) and quantitatively examined using the flow cytometer (FACSCalibur, BD Biosciences, Franklin Lakes, NJ). For investigation of the cellular uptake mechanisms, cells were pre-treated with various endocytosis inhibitors, including Chlorpromazine, Brefeldin A, NaN_3_, Cytochalasin B, Filipin, and Monensin, for 1 h. Then, the cellular uptake of nanocomplexes was quantitatively determined as above.

### Cell counting kit-8 (CCK-8) assay

The effect of the developed nanocomplexes on the cell growth inhibition was evaluated by the CCK-8 kit (Dojindo, Kumamoto, Japan). The transfected HCT-15 cells were seeded in 96-well plates as above and allowed to culture for overnight. Then, the cells were incubated under standard condition for 12, 24, and 48 h, respectively. After that, 10 µL of CCK-8 solution was added into each well and continued to incubate with cells for 1 h. After that, the absorbance was detected using the microplate reader (Thermo Multiskan MK3, Waltham, MA).

### Cell apoptosis assay

The ability of nanocomplexes to induce cell apoptosis was determined using the FITC Annexin V/PI double staining method. The transfected HCT-15 cells were seeded in six-well plates at the density of 1 × 10^4^ cells/well. After 24 h of incubation, the cells were harvested by centrifugation at 1000×*g* for 5 min and then stained with FITC Annexin V Apoptosis Detection Kit I (Becton Dickinson Medical Devices, Shanghai, China). For quantitative analysis, the cells were examined through a FACSscan Flow Cytometer (BD PharMingen, Heidelberg, Germany).

### Western blotting

Total protein samples in cancer cells or tissues were extracted by the RIPA lysis buffer (Beyotime Biotechnology, Shanghai, China) followed by detection of protein concentration using the BCA kit. Then the obtained protein samples were separated by 10% sodium dodecyl sulfate polyacrylamide gel electrophoresis (SDS-PAGE). After the samples were transferred to PVDF membranes, 5% skim milk was added and co-incubated with samples for blocking. Then, primary antibodies were introduced and co-incubated with samples for overnight at 4 °C followed by reaction with horseradish peroxidase-labeled secondary antibodies for 1 h. The proteins levels in cells or tissues were determined using the Bio-Rad ChemiDocTM XRS system (Hercules, CA) with the β-actin was acted as the internal reference.

### Real-time RT-PCR

Total RNA in cancer cells or tissues was obtained using the Trizol reagent (Invitrogen, Carlsbad, CA) and the concentration was determined by Nanodrop Spectrophotometer (ND-2000, Thermo, Waltham, MA). Then, the reverse transcription (RT) reaction of the miRNA and the PCR reactions were respectively performed by PrimeScript RT Master Mix (Perfect Real Time; Takara Bio Inc., Tokyo, Japan) and SYBR Premix ExTaq kit (Takara Bio, Inc., Tokyo, Japan). The expression of RNA was examined using the 2^–ΔΔCt^ approach and normalized to the GAPDH.

### Pharmacokinetic study and biodistribution

SD rats were randomly grouped (*n =* 6) and respectively i.v. injected with pcDNA3.1 + miR-123-3p, PDMAEMA-NP, and CPDMAEMA-NP. For convenient detection, the miR-128-3p was labeled with Cy5. Then, the blood samples were respectively collected at 0.5, 1, 2, 3, 4, 6, 8, and 10 h after injection. The drug concentrations were finally determined by the liquid chromatography–tandem mass spectrometry (LC–MS/MS) analysis.

To investigate the biodistribution of the miR-128-3p formulations, the randomly grouped tumor-bearing mice (*n* = 6) were treated as above. At 24 h after the drug administration, the mice were euthanatized with tumors and primary organs (heart, kidneys, liver, spleen, and lung) were collected. For quantitative evaluation, the drug concentration in tissues, the obtained tumors and organs were homogenized in threefold volumes of distilled water and finally subjected to LC–MS/MS analysis.

### Tumor growth inhibition

The randomly grouped HCT-15 tumor-bearing mice (*n* = 10) were respectively treated with pcDNA3.1 + NC, pcDNA3.1 + miR-123-3p, PDMAEMA-NP, and CPDMAEMA-NP plus with 5-Fu or not. Then, the changes of tumor volume and survival time of mice in each group were carefully observed. The Kaplan–Meier survival curve of mice bearing HCT-15 tumor post various treatments was analyzed using the software Prism (version 5.0). After that, the tumors were obtained for Tdt-mediated dUTP nick-end labeling (TUNEL) assay to detect cell apoptosis and hematoxylin–eosin (H&E) staining to evaluate the toxicity to normal organs.

### Statistical analysis

Data are means ± standard deviation (SD). Comparisons between groups were made via *t*-tests and ANOVAs as appropriate, using GraphPad Prism v7.0 (GraphPad, San Diego, CA). *p*<.05 was the threshold of significance.

### The miR-128-3p was negatively expressed in CRC tissues and cells

**Figure 1.**
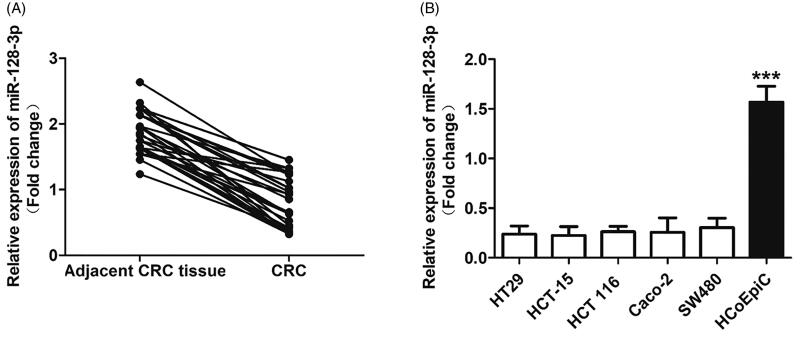
The expression of miR-128-3p significantly down-regulated in the CRC tissues and cells. (A) The levels of miR-128-3p in CRC tissues from 32 patients were quantitatively determined by the RT-qPCR experiments. (B) Quantitatively analysis of the miR-128-3p expression in a series of colorectal cancer cells, including HT29, HCT-15, HCT 116, Caco-2, and SW480 cells, and compared with the normal colorectal epithelial cells HCoEpiC. ***p<.001 vs. the colorectal cancer cells.

The expressions of miR-128-3p in CRC tissues from 32 patients were quantitatively determined by the RT-qPCR experiments. As demonstrated in Figure 1(A), obvious lower expression of miR-128-3p was detected in CRC than that in the adjacent tissues. For further confirmation, the miR-128-3p expression was also investigated in the CRC cells and normal control cells, respectively. As shown in Figure 1(B), the activity of miR-128-3p was dramatically inhibited in the HCT-15 cells. In contrast, the expression of miR-128-3p was signally up-regulated in the normal colorectal epithelial cells HCoEpiC. To further verify, other CRC cells were also negatively expressed with miR-128-3p, the levels of miR-128-3p were respectively determined in HT29, HCT 116, Caco-2, and SW480 cells as well. Not surprisingly, obvious low miR-128-3p expression was detected in the above CRC cells.

The contradictory expression of miR-128-3p in CRC and the normal corresponding tissues or cells indicated that such miRNA may play unfavorable effect on the progress of CRC.

### Characterization of the developed nanocomplexes

**Figure 2.**
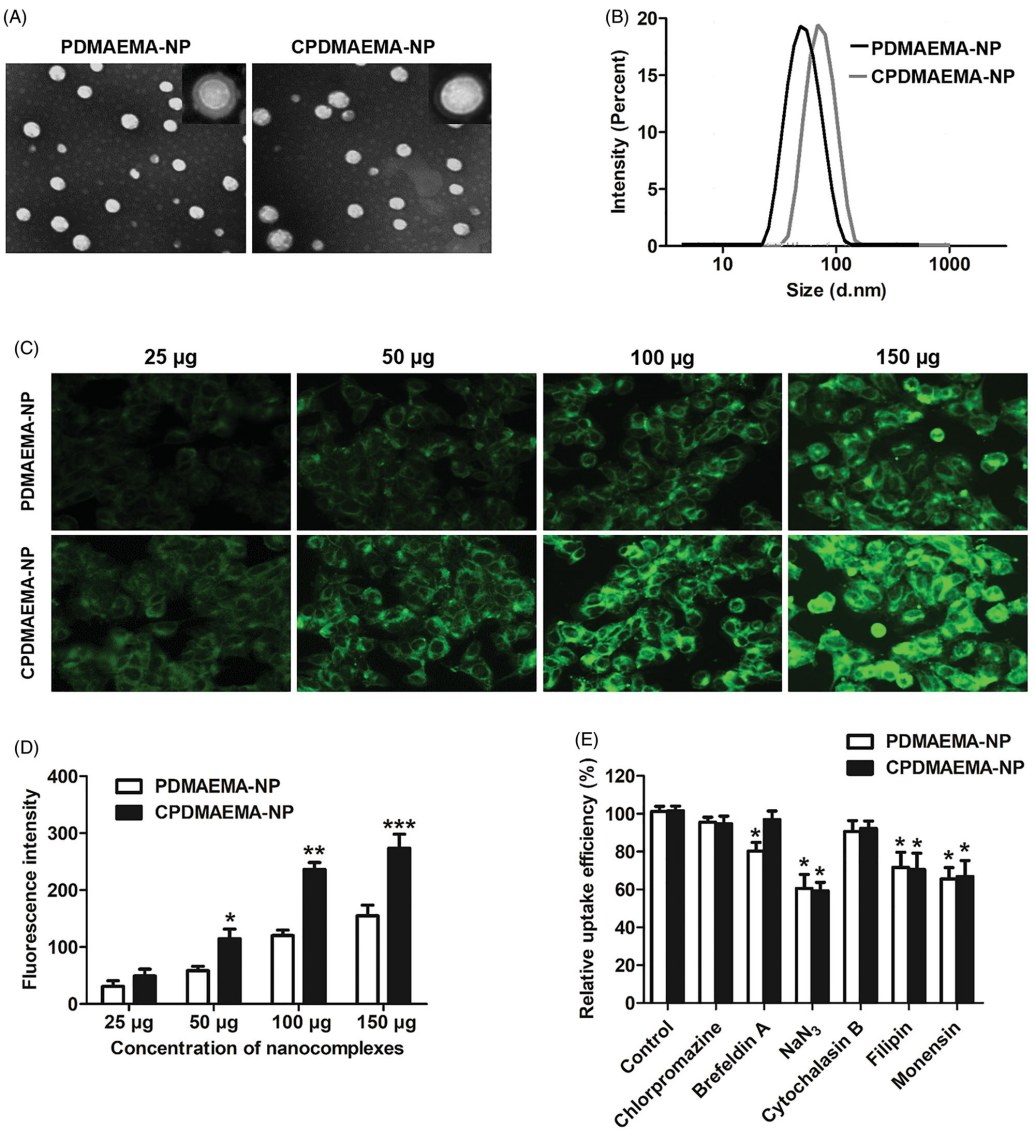
Characterization and cellular uptake of PDMAEMA-NP and CPDMAEMA-NP. (A) Morphology of the developed nanocomplexes by TEM analysis after negatively stained with sodium phosphotungstate solution. (B) DLC evaluation of particle size distribution of PDMAEMA-NP and CPDMAEMA-NP. (C) Qualitative investigation of cellular uptake of PDMAEMA-NP and CPDMAEMA-NP *in vitro* by the fluorescence microscope. (D) Quantitative evaluation of cellular internalization of PDMAEMA-NP and CPDMAEMA-NP *in vitro* by the Flow cytometer. *p<.05, **p<.001, ***p<.001 vs. the cells treated by PDMAEMA-NP. (E) Investigation of the cellular uptake mechanism by pre-treating cells with various endocytosis inhibitors for 1 h. *p<.05 vs. the control group.

As observed under the TEM, both PDMAEMA-NP and CPDMAEMA-NP exhibited the spherical shape and core–shell structure (Figure 2(A)). Additionally, the developed nanocomplexes displayed homogeneous distribution with no obvious aggregations were observed. For DLC analysis, results revealed that the average particle size of PDMAEMA-NP was 79.94 nm. After decoration with C peptides, the particle size was slightly increased to 87.26 nm. Importantly, both nanocomplexes have narrow size distribution with the polydispersity index were 0.123 for PDMAEMA-NP and 0.184 for CPDMAEMA-NP, respectively (Figure 2(B)). Besides, the zeta potentials were further determined, with the PDMAEMA-NP exhibited a value of –23.17 mV while CPDMAEMA-NP has the value of –27.23 mV.

### Cellular uptake of nanocomplexes

As shown in Figure 2(C), cellular uptake of nanocomplexes exhibited the concentration-dependent manner, with the fluorescent intensity increase along with increase of the nanocomplexes concentration. Additionally, more amount of CPDMAEMA-NP was accumulated in HCT-15 cells than PDMAEMA-NP at each nanocomplexes concentration. Such results were further confirmed by the quantitative analysis as displayed in Figure 2(D), indicated that the C peptide was able of mediating more nanocomplexes accumulated in HCT-15 cells. The mechanism of cellular uptake was subsequently investigated by pre-treating cells with various endocytosis inhibitors. As results shown in Figure 2(E) displayed that cellular uptake of PDMAEMA-NP and CPDMAEMA-NP were both signally blocked by NaN_3_, Filipin, and Monensin, suggested that the cellular association of the nanocomplexes was involved with caveolae, energy, and lysosome. Furthermore, pre-treating cells with Brefeldin A markedly inhibited cellular uptake of PDMAEMA-NP while not the CPDMAEMA-NP, indicated the actin was also involved in the process of cell internalization of PDMAEMA-NP but not the CPDMAEMA-NP.

### Investigation of cell transfection efficacy

**Figure 3.**
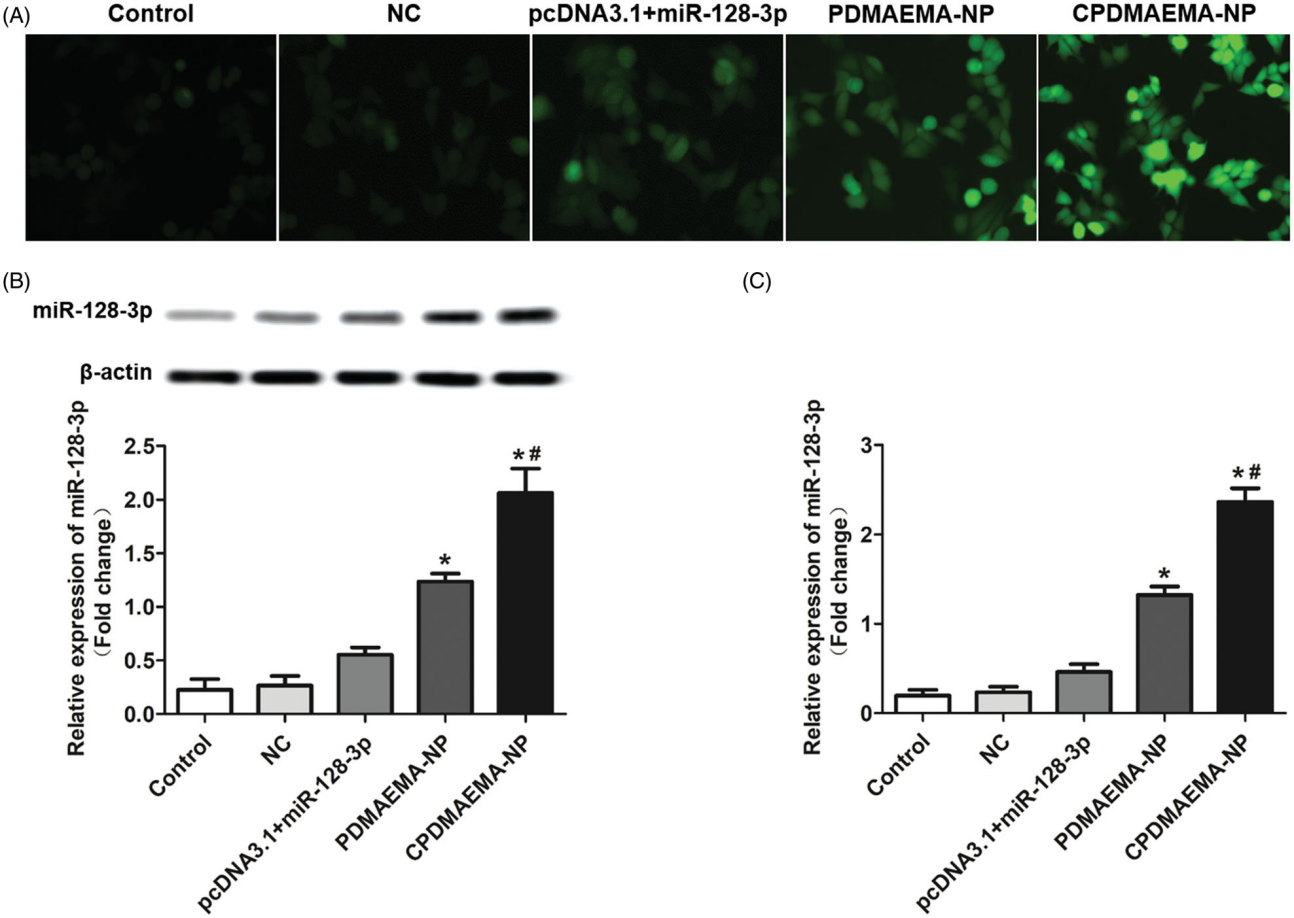
Treating cells with the nanocomplexes developed here resulted higher transfection efficacy than the plasmid mediated approach. (A) Qualitative analysis of the cell transfection efficacy with the fluorescent dye-labeled miR-128-3p was acted as the probe. (B) Qualitative and semi-quantitative evaluation of the miR-128-3p expression in HCT-15 cells by Western blot assay. (C) Quantitative investigation of the miR-128-3p level in HCT-15 cells by RT-qPCR experiments. *p<.05 vs. the control group or NC group. ^#^*p*<.05 vs. the PDMAEMA-NP group.

As shown in Figure 3(A), cells in the group of PDMAEMA-NP exhibited stronger fluorescent intensity compared with the cells treated by pcDNA3.1 + miR-123-3p (Figure 3(A)). Moreover, after decoration with C peptides, the fluorescent signal was further dramatically enhanced due to the ability of C peptides to mediate more accumulation of nanocomplexes within cells. The transfection efficacy was further confirmed by Western blot assay (Figure 3(B)) and RT-qPCR experiments (Figure 3(C)). As confirmed that the cells treated by CPDMAEMA-NP exhibited the highest level of miR-128-3p. Importantly, the expression of miR-128-3p in cells that treat by pcDNA3.1 + miR-123-3p was significantly lower than that in the cells incubated with nanocomplexes. These results together indicated that transfection with cells by the nanocomplexes developed here was superior to the traditional plasmid mediated approach.

### Up-regulation of miR-128-3p significantly inhibited the progress of HCT-15 cells and enhanced the chemotherapy effect of 5-Fu

**Figure 4.**
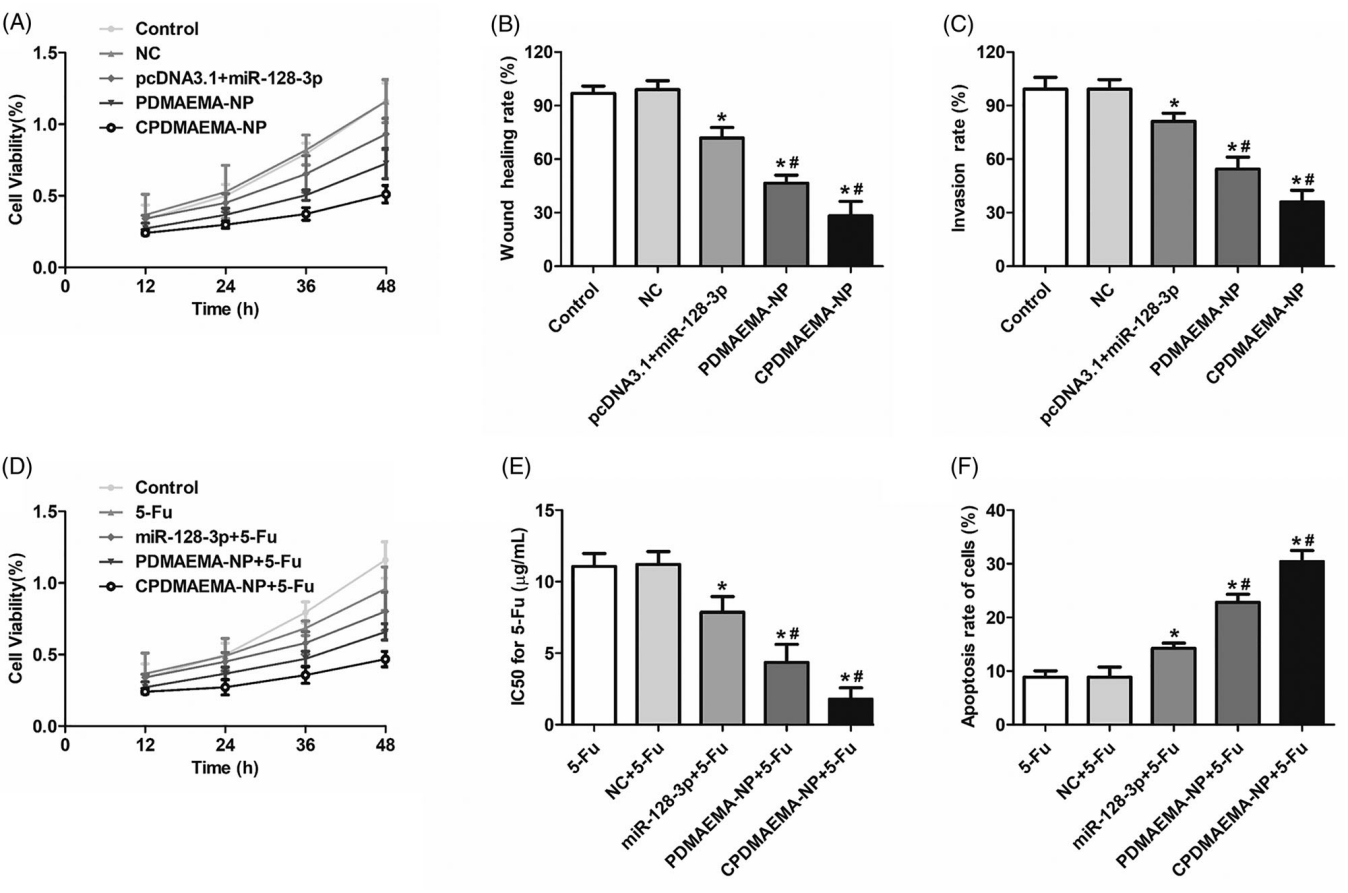
High level of miR-128-3p was adverse to the progress of HCT-15 cells and favorable for the chemotherapy of 5-Fu. (A) Cell viability of HCT-15 cells at different time points after respectively treated by NC, pcDNA3.1 + miR-128-3p, PDMAEMA-NP, and CPDMAEMA-NP. (B) Migration of HCT-15 cells evaluated by Wound healing assay after received various treatments. *p<.05 vs. the control group or NC group. ^#^*p*<.05 vs. the pcDNA3.1 + miR-128-3p group. (C) Invasion of HCT-15 cells evaluated by Trans-well experiment after received various treatments. *p<.05 vs. the control group or NC group. ^#^*p*<.05 vs. the pcDNA3.1 + miR-128-3p group. (D) Cell viability of HCT-15 cells at different time points after respectively treated by 5-Fu, miR-128-3p + 5-Fu, PDMAEMA-NP + 5-Fu, and CPDMAEMA-NP + 5-Fu. (E) IC50 values for 5-Fu after 48 h of treatments. *p<.05 vs. the 5-Fu group or NC + 5-Fu group. ^#^*p*<.05 vs. the miR-128-3p + 5-Fu group. (F) Cell apoptosis rate of HCT-15 cells after respectively treated by 5-Fu plus different miR-128-3p formulations. *p<.05 vs. the 5-Fu group or NC + 5-Fu group. ^#^*p*<.05 vs. the miR-128-3p + 5-Fu group.

As shown in Figure 4(A), the cell growth rate was dramatically down-regulated by transfection with miR-128-3p. Additionally, the inhibition effect of miR-128-3p was signally enhanced after loaded with PDMAEMA nanocomplexes and further enhanced by decoration with C peptides. The migration and invasion ability of HCT-15 cells was respectively determined by Wound healing assays (Figure 4(B)) and trans-well experiments (Figure 4(C)). As results exhibited that cells treated by nanocomplexes have the obvious lower ability of migration and invasion than the pcDNA3.1 + miR-128-3p. Moreover, modification of PDMAEMA-NP with C peptides markedly improved the inhibition effect of miR-128-3p.

**Figure 5.**
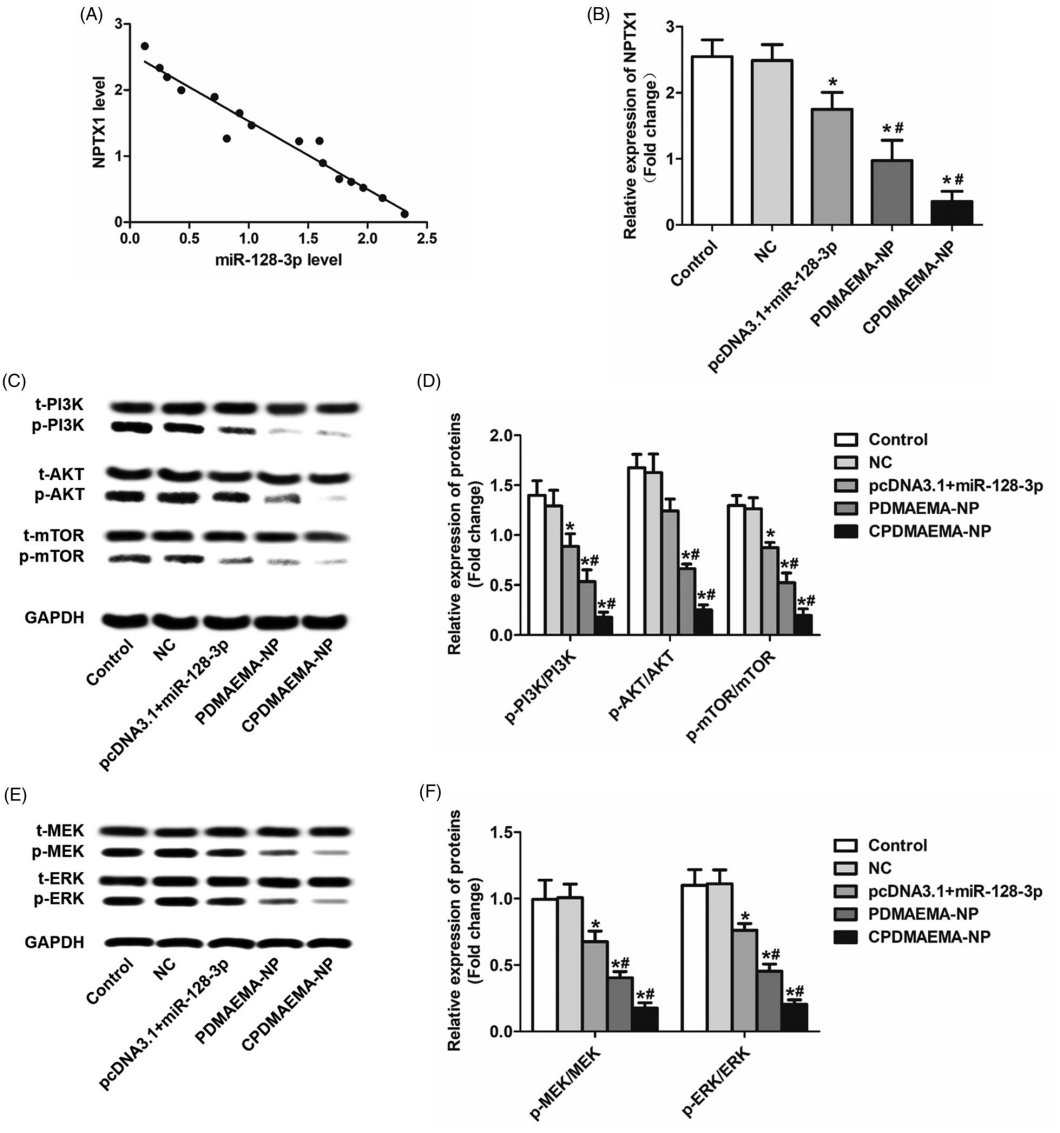
Increase the level of miR-128-3p signally inhibited the activity of PI3K/AKT and MEK/ERK pathway. (A) Qualitative analysis of the expressions of p-PI3K, p-AKT, and p-mTOR by Western blot assays. (B) Quantitative evaluation of the levels of p-PI3K, p-AKT, and p-mTOR through RT-qPCR experiments. (C) Western-blot analysis of the levels of p-MEK and p-ERK in HCT-15 cells after treated by various strategies. (D) The changes of expressions of p-MEK and p-ERK in HCT-15 cells quantitatively analyzed by RT-qPCR experiments. *p<.05 vs. the control group or NC group. ^#^*p*<.05 vs. the pcDNA3.1 + miR-128-3p group.

Further studies revealed that co-treating HCT-15 cells with miR-128-3p formulations plus 5-Fu could achieve obvious lower cell viability compared with the cells treated by 5-Fu alone (Figure 4(D)). The IC50 values for 5-Fu shown in Figure 4(E) indicated that incubation cells with 5-Fu plus miR-128-3p resulted in more sensitivity of HCT-15 cells to the treatment of 5-Fu. Additionally, delivery of miR-128-3p by nanocomplexes signally enhanced the inhibition effect on cells growth. Such effect was further confirmed by the cell apoptosis investigation, with cells in the group of nanocomplexes plus 5-Fu exhibited higher apoptosis rate than that in the group of miR-128-3p + 5-Fu. More importantly, functionalization with C peptides further dramatically enhanced the cytotoxicity to HCT-15 cells (Figure 5(F)).

### miR-128-3p simultaneously silences the PI3K/AKT and MEK/ERK pathway through targeting the neuronal pentraxin 1(NPTX1)

The underlying mechanisms of miR-128-3p regulating the cellular activity of HCT-15 was subsequently investigated. As demonstrated in Figure 5(A), elevation of the miR-128-3p level in HCT-15 cells resulted in down-regulation of NPTX1 and a negative correlation was observed between the expression of miR-128-3p and NPTX1. Moreover, the stronger ability of PDMAEMA-NP than free miR-128-3p and/or miR-128-3p loaded by plasmids to decrease the NPTX1 level was further confirmed by the RT-qPCR analysis (Figure 5(B)). As previous report revealed that NPTX1 plays pivotal role in the development and progress of CRC through activation of the PI3K/AKT pathway (Huo et al., 2019). In this case, the effect of miR-128-3p on the regulation of PI3K/AKT pathway was subsequently investigated. As shown in Figure 5(C), increase the miR-128-3p level signally decreased the expressions of p-PI3K, p-AKT, and p-mTOR. Moreover, treating cells with PDMAEMA-NP resulted more evident decline of the signal of PI3K/AKT pathway. Not unexpectedly, the down-regulation effect of PDMAEMA-NP was signally amplified after decoration with C peptides on its surface. These results were further confirmed by the quantitative analysis of RT-qPCR (Figure 5(D)). In addition to the PI3K/AKT pathway, it was also demonstrated that targeting delivery of miR-128-3p to HCT-15 cells dramatically inhibited the activity of MEK/ERK pathway by signally decreasing the p-MEK and p-ERK expression (Figure 5(E,F)). These results together suggested that the miR-128-3p was able of simultaneously silencing the PI3K/AKT and MEK/ERK pathway by targeting the NPTX1 and the effect could be signally amplified by the CPDMAEMA-NP.

### Tumor targeting delivery of miR-128-3p

**Figure 6.**
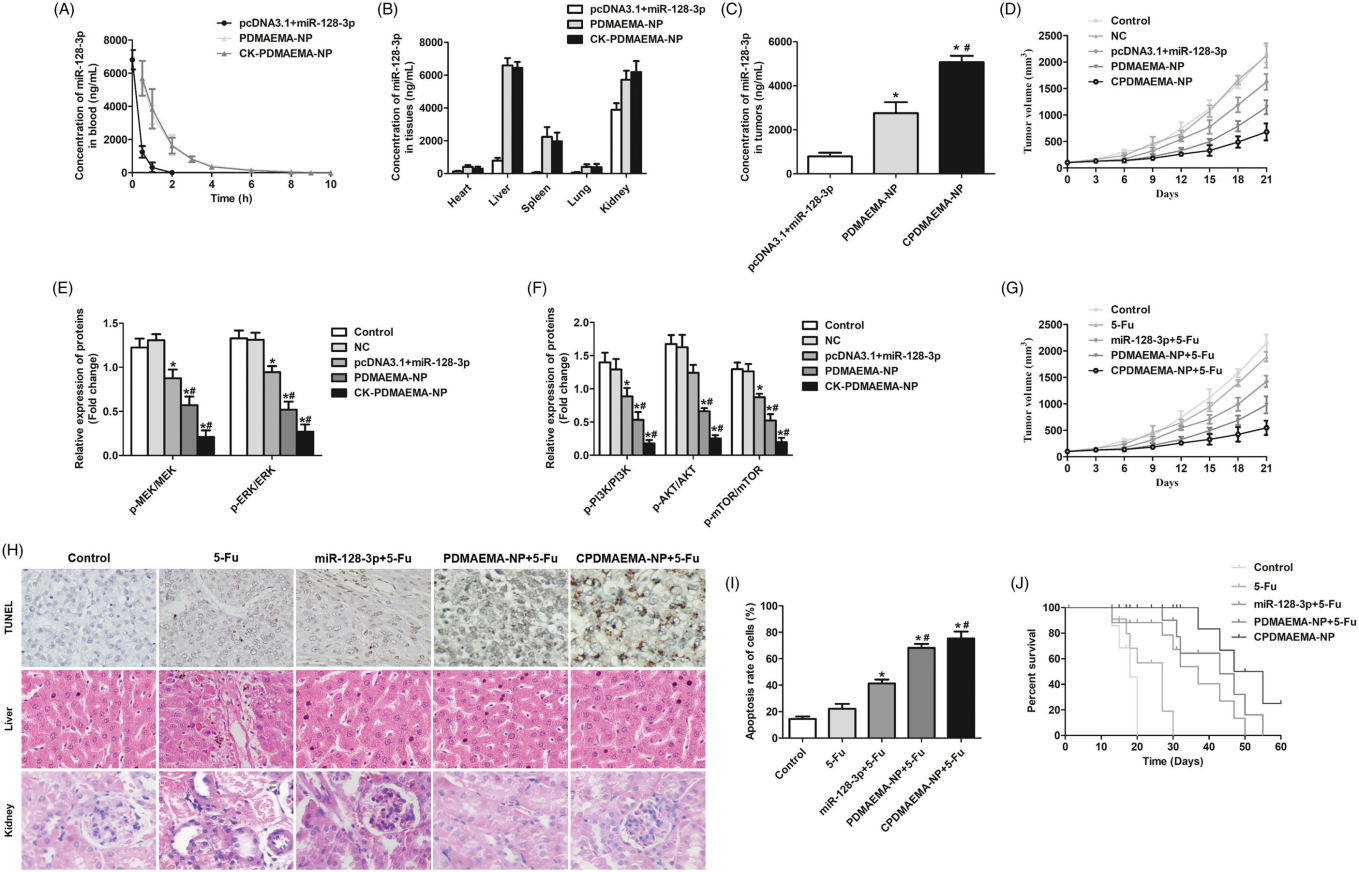
Pharmacokinetics, tumor targeting efficacy, and anti-tumor effect of various miR-128-3p formulations. (A) Evaluation of the pharmacokinetics of pcDNA3.1 + miR-128-3p, PDMAEMA-NP, and CPDMAEMA-NP. Distribution of different miR-128-3p formulations in normal tissues (B) and HCT-15 tumors (C). *p<.05 vs. the pcDNA3.1 + miR-128-3p group. ^#^*p*<.05 vs. the PDMAEMA-NP group. (D) Tumor growth curve of mice after respectively treated with NC, pcDNA3.1 + miR-128-3p, PDMAEMA-NP, and CPDMAEMA-NP. (E) Quantitative evaluation of the levels of p-PI3K, p-AKT, and p-mTOR in tumor tissues by RT-qPCR experiments. *p<.05 vs. the control group or NC group. ^#^*p*<.05 vs. the pcDNA3.1 + miR-128-3p group. (F) The changes of expressions of p-MEK and p-ERK in HCT-15 tumors quantitatively analyzed by RT-qPCR experiments. *p<.05 vs. the control group or NC group. ^#^*p*<.05 vs. the pcDNA3.1 + miR-128-3p group. (G) Tumor growth curve of mice after respectively treated with 5-Fu or 5-Fu plus different miR-128-3p formulations. (H) The area of cell apoptosis after various treatments determined by TUNEL assay and H&E staining of Liver and kidney. (I) Semi-quantitative evaluation of the cell apoptosis rate after various treatments. *p<.05 vs. the control group or 5-Fu group. ^#^*p*<.05 vs. the miR-128-3p + 5-Fu group. (J) Medium survival time of HCT-15 tumor-bearing mice after respectively injected with different formulations.

Before evaluation of the tumor targeting efficacy of CPDMAEMA-NP, the pharmacokinetics of each miR-128-3p formulation was studied. As demonstrated in Figure 6(A), the signal of miR-128-3p in the mice treated by pcDNA3.1 + miR-128-3p was rapidly declined within 2 h. Nevertheless, the circulation time of miR-128-3p was significantly prolonged after delivered by PDMAEMA nanocomplexes. Besides, similar metabolic trend was observed between the PDMAEMA-NP and CPDMAEMA-NP, indicated the modification with peptides did not affect the properties of nanocomplexes.

Tumor targeting efficacy was subsequently investigated in the HCT-15 tumor-bearing mice. As shown in Figure 6(B,C), weak signal of miR-128-3p was detected in most of the normal tissues (heart, liver, spleen, and lung) and tumors, confirmed the quick elimination feature of pcDNA3.1 + miR-128-3p. In contrast, the mice treated with PDMAEMA-NP not only achieved more accumulation of miR-128-3p in liver, spleen, and kidney, but also more amount of miR-128-3p in tumor tissues. Additionally, the delivery efficacy of miR-128-3p by PDMAEMA-NP could be further markedly enhanced by decoration with C peptides.

### Tumor growth *in vivo*

The role of miR-128-3p in regulation of tumor growth was further investigated *in vivo*. As illustrated in Figure 6(D), rapidest increase trends of tumor volumes were observed in the group of control and NC. However, the growth trend was signally down-regulated after treated by pcDNA3.1 + miR-128-3p, indicated the negative regulation effect of miR-128-3p on the progress of HCT-15 tumor. Of great importance, the tumor growth inhibition effect of miR-128-3p could be memorably amplified after delivered by PDMAEMA-NP. Moreover, such anti-tumor effect was further dramatically enhanced after decoration with the tumor-homing C peptides. Similar to the cellular experiments, the miR-128-3p negatively regulated the progress of HCT-15 tumor *in vivo* through decrease the levels of p-PI3K, p-AKT, p-mTOR, p-MEK, and p-ERK (Figure 6(E,F)). It indicated that the PI3K/AKT and MEK/ERK pathways were both inhibited by miR-128-3p.

The effect of 5-Fu plus miR-128-3p on combating the progress of HCT-15 tumor was further studied. As demonstrated in Figure 6(G), obvious lower increase rate of tumor volume was obtained in the mice treated by 5-Fu plus miR-128-3p than the mice only injected with 5-Fu. Additionally, co-treating the mice with 5-Fu and nanocomplexes achieved the more satisfactory inhibition effect, which contributed to larger area of cell apoptosis in tumor tissues than other groups (Figure 6(H,I)). More importantly, in addition to the improved anti-tumor effect, delivery of miR-128-3p by the developed nanocomplexes also leaded to lower toxicity to the main normal metabolic organs (liver and kidney). In contrast, obvious serious and mild cellular damage was detected in the metabolic organs of the mice respectively treated by 5-Fu and miR-128-3p plus 5-Fu (Figure 6(I)). Finally, the survival time of HCT-15 tumor-bearing mice after different treatments was investigated. As expected, the mice treaded with 5-Fu plus miR-128-3p achieved the longer medium survival time than the mice only injected with 5-Fu (Figure 6(J)). Furthermore, the survival time of 5-Fu + miR-128-3p group could be signally prolonged by delivery with PDMAEMA-NP and further improved by CPDMAEMA-NP.

## Discussion

Increasing evidence revealed that many kinds of miRNAs were involved in a wide range of biological processes as functioned as tumor suppressor genes or oncogenes through regulation of multiple target genes levels (Kushlinskii et al., 2016; Kager et al., 2017). MiR-128-3p, has significant role in speeding up of cell cycle arrest and chromosomal instability, was demonstrated to be an oncogene in malignancies such as acute leukemia, breast cancer, and lung cancer (Cai et al., 2017; Block et al., 2018; Guo et al., 2018). Nevertheless, tumor suppressive functions of miR-128-3p were also proved in other cancer types such as the human esophageal squamous-cell carcinoma and hepatoma carcinoma cell (Yao et al., 2015; Guo et al., 2018). In the present study, scarce expression of miR-128-3p was detected in CRC tissues obtained from 32 patients with late stage of CRC. Additionally, insufficient activity of miR-128-3p was further confirmed in a series of CRC cells while obvious higher level of miR-128-3p was detectable in the corresponding normal cells. The results preliminarily indicated the negative role of miR-128-3p in the developed of CRC. Such hypothesis was confirmed by our further studies with increasing the levels of miR-128-3p significantly inhibited the proliferation, migration, and invasion of CRC cells and progress of CRC tissues. Moreover, the up-regulation of miR-128-3p also improved the sensitivity of CRC cells or tissues to 5-Fu treatment.

The miRNAs promotes or inhibition tumorigenesis and metastasis mainly through regulation of the gene mutation and/or abnormal activation of various signal pathways (Kager et al., 2017). NPTX1 has been reported to be intimately associated with a wide range of cancers and its aberrant activation was closely related to tumorigenesis and progress (Mori et al., 2011). Our studies revealed that elevation of miR-128-3p signally down-regulated the NPTX1 levels in CRC cells. PI3K/AKT has been demonstrated to be one of the most pivotal regulator of tumorigenesis and progress of many cancer types (Faes & Dormond, 2015). Previous studies have proved that excessive activation of the PI3K/AKT pathway was closely related to proliferation, migration, and invasion of tumor cells (Alqurashi et al., 2018). It was reported that the NPTX1 regulated the development and growth of cancers mainly through activation of the PI3K/AKT pathway (Huo et al., 2019). In the present study, the effect of miR-128-3p on the regulation of PI3K/AKT signaling was therefore investigated. As demonstrated that up-regulation of the miR-128-3p levels resulted in silence of PI3K/AKT signal in CRC cells and tissues. In contrast, insufficient expression of miR-128-3p in the untreated CRC cells leaded to abnormal activation of PI3K/AKT pathway, which in turn contributed to high rate of proliferation, migration, and invasion. Moreover, our studies also revealed that the activity of MEK/ERK pathway was simultaneously inhibited after elevation of the miR-128-3p levels. The MEK/ERK represents another critical signaling pathway in colorectal and melanoma cancer progression by regulating cancer cell proliferation and survival (Liu et al., 2014). Based on this, the miR-128-3p could be the dual MEK-ERK and PI3K-AKT cell signaling pathway inhibitor through targeting the NPTX1.

Although the miRNA has been attractive for therapeutic use due to its high efficiency and easily synthesized, the unstable properties and low circulation half-life significantly impaired the widely application (Rzepiel et al., 2019). As demonstrated in the present study, delivery of miR-128-3p by plasmid exhibited extremely instable *in vivo* with a rapid metabolism within 2 h. To avoid premature degradation and prolong the circulation time of miRNAs in bloodstream, the miRNAs should be shield enough and escaped from filtered out by kidney. In our study, to achieve that goal, the miR-128-3p was loaded into the inner core of PDMAEMA-NP. By this way, the miR-128-3p was shielded from exposure to various degrading enzymes in bloodstream. Moreover, the terminal of PDMAEMA was functionalized with PEG chains, which was favorable for long time of circulation by minimizing nonspecific interactions of nanoparticles with serum proteins (Fernandez-Piñeiro et al., 2017). Pharmacokinetics investigation revealed that the circulation time of miR-128-3p was dramatically prolonged after it was delivered by PDMAEMA nanocomplexes.

Recently, ligand-based active targeting strategy has been widely developed for its excellent capacity of improving drug delivery efficiency (Bazak et al., 2015). By utilizing these specific ligands, which are able of targeting specific receptors that were over-expressed on cancer cells, the accumulation of drugs in tumor tissues could be signally enhanced and unwanted side-effects could be markedly down-regulated as well (Zhong et al., 2019). The C peptide, with the sequence of CPKSNNGVC, was a recently developed peptide designed to specifically target the MCT1 (Ferreira et al., 2019). MCT1 plays significant role in catalyzing of the proton-linked movement of many monocarboxylates and promotion of lactate influx into tumor cells, which finally contributed to tumor metabolism (Benjamin et al., 2018). Additionally, increased MCT1 expression was closely related to the disease progression and prognosis in a wide range of human malignancies (Miranda-Gonçalves et al., 2013). Previous studies have demonstrated that aberrant expression of MCT1 was detectable in CRC while not the normal tissues (Pinheiro et al., 2008). Based on this, the MCT1 might be an ideal target for realize tumor targeting therapy. In our study, the surface of PDMAEMA-NP was decorated with the C peptide. Both *in vitro* and *in vivo* experiments demonstrated that modification of C peptide markedly enhanced CRC cells and/or tissues accumulation of miR-128-3p, which in turn leaded to the strongest anti-tumor effect. Moreover, the C peptides mediated drug delivery also leaded to more safety to the normal organs mainly due to largely reduced the unwanted accumulation of drugs at the nonactive sites.

## Conclusions

In summary, the present study demonstrated that elevation of the miR-128-3p level resulted in obvious inhibition effect on the growth, migration, and invasion of CRC. Further underlying mechanisms studies revealed that miR-128-3p down-regulated the deteriorate rate of CRC through simultaneously silencing the activity of PI3K/AKT and MEK/ERK pathway. To safely and efficiently deliver the miR-128-3p *in vivo*, miR-128-3p was loaded in to the developed PDMAEMA-NP and decorated with CRC-homing peptide for active targeting therapy. The results confirmed that the CPDMAEMA-NP not only contributed to the most efficient anti-tumor effect, but also leaded to the least toxic side effects.
